# Consecutive assessment of FA and ADC values of normal lumbar nerve roots from the junction of the dura mater

**DOI:** 10.1186/s12891-015-0576-4

**Published:** 2015-06-27

**Authors:** Ryo Miyagi, Toshinori Sakai, Eiko Yamabe, Hiroshi Yoshioka

**Affiliations:** Department of Orthopedics, The University of Tokushima Graduate School, Tokushima, Japan; Department of the Radiological Sciences, University of California, Irvine, School of Medicine, Irvine, CA 92868 USA

**Keywords:** Diffusion weighted imaging (DWI), Diffusion tensor imaging (DTI), Fractional anisotropy (FA), Apparent diffusion coefficient (ADC)

## Abstract

**Background:**

Diffusion weighted imaging (DWI) and diffusion tensor imaging (DTI) are widely used in the evaluation of the central nervous system and recently have been reported as a potential tool for diagnosis of the peripheral nerve or the lumbar nerve entrapment. The purpose of this study was to evaluate consecutive changes in apparent diffusion coefficient (ADC) and fractional anisotropy (FA) values of normal lumbar nerve roots from the junction of the dura mater.

**Methods:**

The lumbar spinal nerves were examined in 6 male healthy volunteers (mean age, 35 years) with no experiences of sciatica, with a 3.0-T MR unit using a five-element phased-array surface coil. DTI was performed with the following imaging parameters: 11084.6/73.7 ms for TR/TE; b-value, 800 s/mm2; MPG, 33 directions; slice thickness, 1.5 mm; and total scan time, 7 min 35 s. ADC and FA values at all consecutive points along the L4, L5 and S1 nerves were quantified on every 1.5 mm slice from the junction of the dura mater using short fiber tracking.

**Results:**

ADC values of all L4, 5, and S1 nerve roots decreased linearly up to 15 mm from the dura junction and was constant distally afterward. ADC values in the proximal portion demonstrated S1 > L5 > L4 (p < 0.05). On the other hand, FA values increased linearly up to 15 mm from the dura junction, and was constant distally afterward. FA values in the proximal portion showed L4 > L5 > S1 (p < 0.05).

**Conclusion:**

Our study demonstrated that ADC and FA values of each L4, 5, and S1 at the proximal portion from the junction of the dura matter changed linearly. It would be useful to know the normal profile of DTI values by location of each nerve root so that we can detect subtle abnormalities in each nerve root.

## Background

Diffusion tensor imaging (DTI) is widely used in the evaluation of the central nervous system and have recently been reported as a potential tool for diagnosis of the peripheral nerve or the lumbar nerve entrapment [1–8]. The index of fractional anisotropy (FA) shows the degree of the anisotropy of the analyzed structure, and apparent diffusion coefficient (ADC) shows the degree of diffusion. Both imaging methods can be deployed as a quantitative appraisal method of a damaged nerve. Some authors reported that entrapped nerve roots in symptomatic patients showed decreased FA values and increased ADC values [4, 5]. FA and ADC values in these reports were compared at discrete proximal and distal parts. There has been no report to evaluate a fine consecutive change of these values. In addition, they are known to be affected by various factors such as the measurement method and partial volume effect. However, there were few reports about measurement methodology [4]. The purpose of this study was to evaluate consecutive changes in apparent diffusion coefficient (ADC) and fractional anisotropy (FA) values of normal lumbar nerve roots from the junction of the dura mater.

## Methods

### Study subjects

The study was approved by the institutional review board of University of California, Irvine, and conformed to the Declaration of Helsinki. Written informed consent was obtained from each subject. The lumbar spinal nerves were examined in 6 male healthy volunteers (mean age, 35 years, range, 32–43 years) with no history of lumbar surgery, neurological findings such as muscle weakness or sensory disturbance, or clinical history of sciatica, with a 3.0-T MR unit (Achieva; Philips Healthcare, Best, The Netherlands) using a five-element phased-array surface coil.

### MRI acquisition

DTI was performed with the following imaging parameters: 11084.6/73.7 ms for TR/TE, respectively; flip angle, 90°; field of view, 280 mm; b-value,800 s/mm^2^; MPG, 33 directions; slice thickness/gap, 1.5 mm; number of slice, 60; actual voxel size, 1.49X2.98X1.50 mm^3^; and total scan time, 7 min 35 s.

### MRI interpretation

**Fig. 1:**
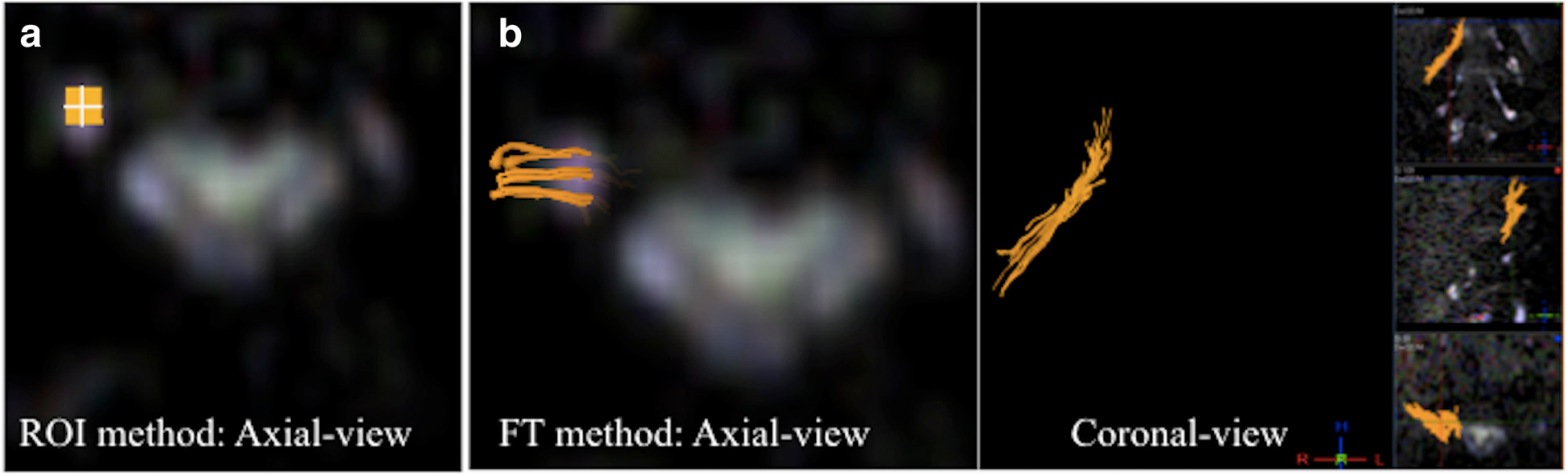
ROI method and FT method for measuring FA and ADC. **a** In the ROI method, a region of interest was placed in expected axial DTI without fiber tracking. To minimize partial volume effects, we set 4 voxels as a ROI in the lumbar spinal nerve. **b** In the FT method using fiber tracking, FA and ADC values were calculated from the drawn fibers passing through the target nerve only in reference to 3 smaller vertical displays of the reformatted coronal and sagittal images as well as original axial images on the right

Mean FA and ADC values at all consecutive points along the L4, L5 and S1 nerves were quantified by two orthopaedic surgeons (RM: 6 years of experience and TS: 20 years of experience) using two methods: direct measurement of FA and ADC values placing a region of interest (ROI) on expected axial images without fiber tracking (ROI method), and with fiber tracking (FT method) [Fig. 1a, b]. Both observers had 10 months of experience in DWI and DTI analysis of the nerve roots. Each observer evaluated FA and ADC values with two methods, twice to measure inter-rater and intra-rater reproducibility. FA and ADC values at all consecutive points along the L4, L5 and S1 nerves were quantified on every 1.5 mm slice from the junction of the dura mater. All measurements were performed on PC workstations using the imaging software Achieva 3 T TX system, release 3.2. On the ROI method, to minimize partial volume effect, we set 4 voxels as a ROI in the lumbar spinal nerve. We confirmed the fibers are passing through only the target nerve using coronal- and sagittal images when using the FT method. The thresholds for tracking termination were 0.1 for FA, 27° for the angle, 3 mm for minimum fiber length.

### Statistical analysis

All FA and ADC values (n = 754/each method) were analyzed by Pearson’s correlation coefficient to detect their concordance rates between the two methods. The associations of the differences of the mean of paired measurements (Bland-Altman methods) were used to examine absolute differences between the two methods. We measured concordance correlation coefficient values (CCC) for all values to define inter-and intra-rater reproducibility by Pearson's Correlation analysis. Each CCC of all 36 nerve roots was evaluated as follows: Excellent (0.9 ≤ CCC), Good (0.75 ≤ CCC < 0.9), Poor (CCC < 0.75). All statistical analyses were performed using SPSS statistical software (IBM SPSS Statistics, IBM Corporation, Chicago, IL). We compared ADC and FA values among the L4, L5 and S1 nerves using the Kruskal—Wallis test. Inter-rater and intra-rater reproducibility for ROI and FT methods was statistically analyzed with Chi-square for independence test. Differences with P < 0.05 were regarded as statistically significant.

## Results

### Comparison of FT and ROI methods

**Fig. 2:**
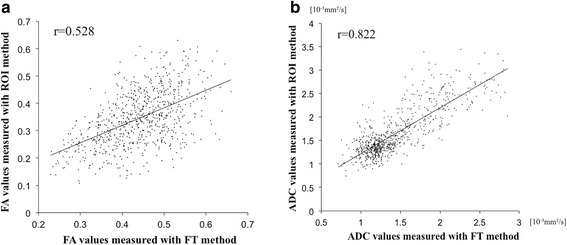
Concordance rates of the two methods. **a** FA measured with FT method versus ROI method were inconsistent (*r* = 0.528). However, **b**) ADC values had a relatively good consistency (*r* = 0.822)

**Fig. 3:**
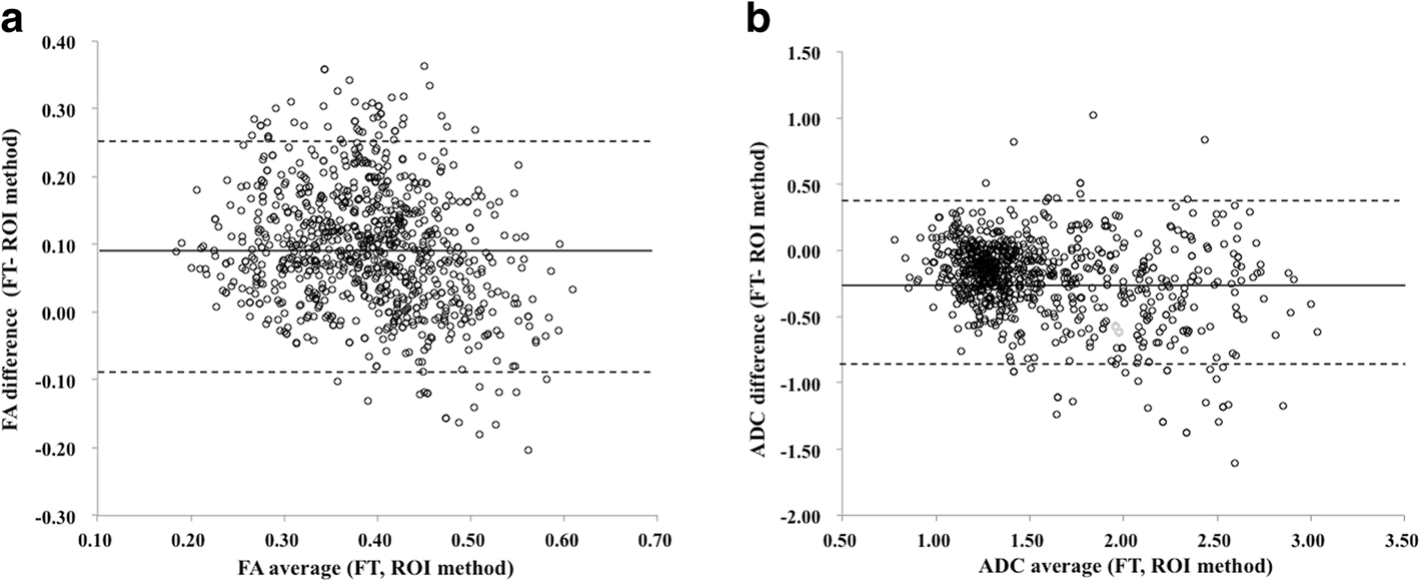
Bland- Altman analysis of the absolute mean differences of FT methods and ROI methods in FA value (**a**) and ADC value (**b**). The solid line represents the mean bias, and the dashed lines represent the limits of agreement(mean ± two standard deviations). In FA value, mean vias and SD of 0.095 ± 0.091 and limits of agreement of −0.087 to 0.277. In ADC value, mean vias and SD of −0.199 ± 0.295 and limits of agreement of −0.789 to 0.391

Concordance rates of the two methods were 0.528 on FA values, and 0.822 on ADC values [Fig. 2a, b]. Bland-Altman analysis identified that absolute mean bias of FA values (0.095 ± 0.182) and ADC values (−0.199 ± 0.590) [Fig. 3a, b]. Table 1 shows the results of mean inter- and intra-rater reproducibility on each nerve root [Table 1]. The reproducibility varied according to the nerve root, with the highest of the S1 nerve. FT method demonstrated significantly better, and almost perfect, inter-rater reproducibility (FA: 100%, ADC: 97.2%) compared with ROI method (FA: 63.9%, ADC: 75.0%) [Table 2]. Also, FT method had a statistically significant excellent/good intra-rater reliability (FA: 91.7%, ADC: 97.2%) compared with ROI method (FA: 69.4%, ADC: 83.3%) on both FA and ADC values [Table 2].

**Table 1:** Inter-rater and Intra-rater reliability of concordance correlation coefficient (CCC) for FA and ADC Measurements

		Inter-rater		Intra-rater	
		FT	ROl	FT	ROl
L4 (N=103)	FA R/L	0.89/0.89	0.68/0.54	0.79/0.83	0.78/0.78
	ADC R/L	0.96/0.93	0.85/0.85	0.87/0.93	0.96/0.86
L5 (N=127)	FA R/L	0.96/0.95	0.65/0.77	0.93/0.93	0.69/0.79
	ADC R/L	0.96/0.96	0.82/0.93	0.96/0.95	0.88/0.90
S1 (N=135)	FA R/L	0.96/0.98	0.85/0.90	0.96/0.95	0.77/0.87
	ADC R/L	0.96/0.93	0.92/0.95	0.98/0.98	0.97/0.96

**Table 2:** Summary of data on inter- and intra-rater reproducibility

		FA		ADC	
		ROI method	FT method	ROI method	FT method
Inter-rater Reproducibility	Excellent/Good	23 (63.9%)		27 (75.0%)	
	Poor	13 (36.1%)		9 (25.0%)	
Intra-rater Reproducibility	Excellent/Good	25 (69.4%)		30 (83.3%)	
	Poor	11 (30.6%)		6 (16.7%)	

Concordance Correlation Coefficient: CCC, Excellent: 0.90≤CCC, Good: 0.75≤CCC<0.90, Poor: CCC<0.75, Chi-square test, *p<0.05

### FA and ADC measurements

**Fig. 4:**
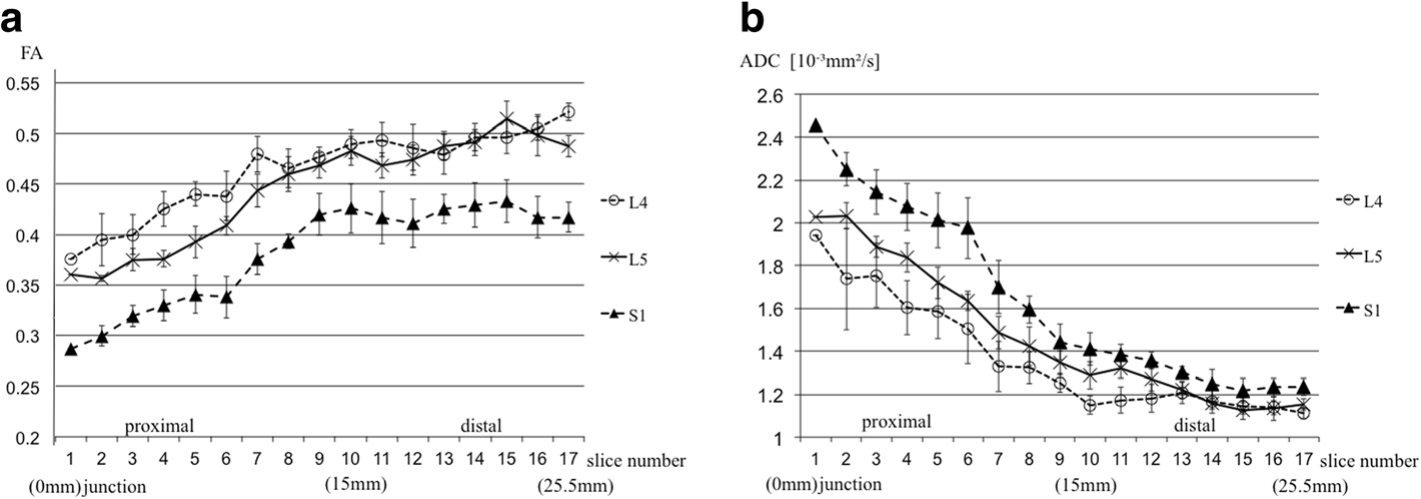
Change in FA and ADC values from the junction of the dura mater. **a** FA values of all L4, L5, and S1 increased linearly up to 15 mm from the dura junction and were constant distally afterward. **b** ADC values of all L4, L5, and S1 nerve roots decreased linearly up to 15 mm from the dura junction and were constant distally afterward

Based on these results we used FT methods to measure FA and ADC values at all consecutive points along the L4, L5 and S1 nerves. FA values of all L4, 5, and S1 nerve roots increased linearly up to 15 mm from the dura junction and was constant distally afterward [Fig. 4a]. FA values in the proximal portion demonstrated a statistically significant trend L4 > L5 > S1 (p < 0.0001). On the other hand, ADC values decreased linearly up to 15 mm from the dura junction, and was constant distally afterward [Fig. 4b]. ADC values in the proximal portion showed a statistically significant trend S1 > L5 > L4 (p < 0.0001).

## Discussion

Diffusion MRI is an imaging modality that enables quantification of medical imaging modality that is able to quantify the anisotropic diffusion of water molecules in biological tissues [9]. DTI has been widely used in the evaluation of the central nervous system [9–18]. FA values show the size of the anisotropy of the analyzed structure by taking advantage of the improved directional evaluation of water diffusivity in abnormal areas. They are generally decreased in the presence of local extracellular edema, or where a reduced number of fibers results in increased extracellular space [19]. The other parameter, ADC, is a scalar value reflecting molecular diffusivity under motion restriction. Demyelination and edema by slow compression result in an increased degree of diffusivity as indicated by increased ADC values compared to those of normal tissue [3]. Recent studies have reported that FA and ADC values could be a potential tool to present the severity of nerve entrapment and useful tool for diagnosis of lumbar nerve entrapment [1–4]. Eguchi et al. reported that mean FA values in entrapped nerve roots were significantly lower than those in intact nerve roots [1]. They also reported increased ADC values in a compressed dorsal root ganglion and distal spinal nerves, values which were thought to reflect an entrapped 11 nerve root due to edema and Wallerian degeneration [2]. Other studies also reported that entrapped nerve roots in symptomatic patients showed decreased FA values and increased ADC values [4, 5]. In these reports, FA and ADC values were compared between the discrete proximal and distal part of the nerve root. However, there has been no report to evaluate fine continuous changes of these values. Our study demonstrated that normal or asymptomatic FA and ADC values of each L4, 5, and S1 were different. FA values in the proximal portion demonstrated a statistically significant trend L4 > L5 > S1. On the other hand, ADC values in the proximal portion showed a statistically significant trend S1 > L5 > L4. They changed linearly in the proximal portion of the nerve root, but were almost constant in the distal portion. These findings suggest importance of recognizing the consecutive normal profile of FA and ADC values of each nerve root in order to detect focal subtle changes. To the best of our knowledge, this is the first study to measure FA and ADC values at all consecutive points of lumbar nerve roots.

These values are known to be affected by various factors such as the measurement method and partial volume effect. In the FT method, we measured FA and ADC using the fiber tracking extended to adjacent several slices from the selected axial slice and the measured values were averaged of those slices. Therefore, these results might not reflect the accurate FA and ADC values at the pinpoint. In the ROI method, FA and ADC values were measured directly at the pinpoint on each slice and not influenced by the adjacent slices. Therefore, the ROI method for quantitation of FA and ADC values seems to be more theoretically accurate than FT method. However, these values in the ROI method were easily influenced by the adjacent more isotropic cerebrospinal fluid. Our study demonstrated that FA values measured with FT methods and ROI methods were inconsistent (*r* = 0.528), while ADC values had a relatively good consistency (*r* = 0.822). Both FA and ADC values measured with the FT method had significantly better reproducibility. Although we set small ROIs over the lumbar spinal nerve to minimize partial volume effect in the ROI method, the results in this study demonstrated low reproducibility with this method. The FT method includes mainly two advantages over the ROI method for measurements of FA and ADC values. One advantage is reduction of time to draw ROIs at each level, and the other is higher reproducibility regardless of less sensitivity to poor contrast between the nerve root and surrounding tissue. Therefore, it is very important to set up threshold parameters of fiber tracking. In this study, we used short fiber tracking to minimize the average effect of the fiber tracking method.

There are some limitations in this study. First, the numbers of normal subjects were small. Second, no symptomatic patients were included in this study to compare normal FA and ADC values with pathological ones. However, the results from all healthy subjects in the current study demonstrated the same trend without exception. Also, our purpose was to establish a more reliable method to analyze FA and ADC before starting a large number of clinical studies. A larger scale clinical study including normal volunteers and symptomatic patients is needed to confirm and further evaluate our findings from a small number of healthy volunteers. Finally, we need further technical improvements such as an increased signal to noise ratio in order to obtain images with higher image quality or higher matrix size.

## Conclusion

Our study demonstrated that ADC and FA values of each L4, 5, and S1 at the proximal portion from the junction of the dura matter changed linearly. It would be useful to know the normal profile of DTI values by location of each nerve root so that we can detect subtle abnormalities in each nerve root.

## Abbreviation

MRI: Magnetic resonance imaging
ROI: region of interest
DWI: Diffusion weighted imaging
MPG: Motion probing gradient
DTI: Diffusion tensor imaging
FA: Fractional anisotropy
ADC: Apparent diffusion coefficient
FT: Fiber tracking
CCC: Concordance correlation coefficient values
